# Supplementary material for: Heat adaptation of phage T7 under an extended genetic code
Source: Virus Evol. 2021 Dec 1;7(2):veab100. doi: 10.1093/ve/veab100 (PMC8923235; doi:10.1093/ve/veab100)
Supplement: veab100_Supp [file veab100_supp.zip › Sequencing_Analysis.zip!/Sequencing_Analysis/S3/evidence/RA_30.html]

BRESEQ :: Evidence


| Read alignment evidence... | | | | | | | | | | | |
| --- | --- | --- | --- | --- | --- | --- | --- | --- | --- | --- | --- |
|  | seq id | position | | ref | new | freq | score (cons/poly) | reads | annotation | genes | product |
| \* | NC\_001604.1 | 28,691 | 0 | A | C | 44.5% | 97.1 / inf | 1448 | intergenic (–/–) | *–/–* | –/– |
| Reads supporting (aligned to +/- strand):  **ref** base A (397/403);  **new** base C (334/308);  **total** (734/714) | | | | | | | | | | | |
| Fisher's exact test for biased strand distribution *p*-value = 3.68e-01 | | | | | | | | | | | |
| Kolmogorov-Smirnov test that lower quality scores support variant *p*-value = 2.00e-01 | | | | | | | | | | | |

Only 100 of 1487 total aligned reads displayed.  

`GGAGGTATGGGCTATCGTGCAGCAACCACTCAGGCCGAACAGCCAAGGTCAAGCCTATTGGACACCATTGGTCGGTTCGCTAAGGCTGGTGCCGATATGTATACCGCTAAGGAACAACGAGCACGAGACCTAGCTGATGAACGCTCTAACGAGATTATCCGTAAGCTGACCCCTGAGCAACGTCGAGAAGCTCTCAACAACGGGACCCTTCTGTATCAGGATGACCCATACGCTATGGAAGCACTCCGAGTCAAGACTGGTCGTAACGCTGCGTATCTTGTGGACGATGACGTTATGCAGAAGATAAAAGAGGG‑TGTCTTCCGTACTCGCGAAGAGATGGAAGAGTATCGCCATAGTCGCCTTCAAGAGGGCGCTAAGGTATACGCTGAGCAGTTCGGCATCGACCCTGAGGACGTTGATTATCAGCGTGGTTTCAACGGGGACATTACCGAGCGTAACATCTCGCTGTATGGTGCGCATGATAACTTCTTGAGCCAGCAAGCTCAGAAGGGCGCTATCATGAACAGCCGAGTGGAACTCAACGGTGTCCTTCAAGACCCTGATATGCTGCGTCGTCCAGACTCTGCTGACTTCTTTGAGA  >  NC_001604.1/28391‑28991`  

                                                                                                                                                                                                                                                                                                            `|`                                                                                                                                                                                                                                                                                                               
  `aGGTATGGGCTATCGTGCAGCAACCACTCAGGCCGAACAGCCAAGGTCAAGCCTATTGGACACCATTGGTCGGTTCGCTAAGGCTGGTGCCGATATGTATACCGCTAAGGAACAACGAGCACGAGACCTAGCTGATGAACGCTCTAACGAGATTATCCGTAAGCTGACCCCTGAGCAACGTCGAGAAGCTCTCAACAACGGGACCCTTCTGTATCAGGATGACCCATACGCTATGGAAGCACTCCGAGTCAAGACTGGTCGTAACGCTGCGTATCTTGTGGACGATGACGTTATGcagcag                                                                                                                                                                                                                                                                                                             <  1:100013/301‑1 (MQ=255)`  
   `ggTATGGGCTATCGTGCAGCAACCACTCAGGCCGAACAGCCAAGGTCAAGCCTATTGGACACCATTGGTCGGTTCGCTAAGGCTGGTGCCGATATGTATACCGCTAAGGAACAACGAGCACGAGACCTAGCTGATGAACGCTCTAACGAGATTATCCGTAAGCTGACCCCTGAGCAACGTCGAGAAGCTCTCAACAACGGGACCCTTCTGTATCAGGATGACCCATACGCTATGGAAGCACTCCGAGTCAAGACTGGTCGTAACGCTGCGTATCTTGTGGACGATGACGTTATGCagagga                                                                                                                                                                                                                                                                                                            >  1:227223/1‑301 (MQ=255)`  
    `gTATGGGCTATCGTGCAGCAACCACTCAGGCCGAACAGCCAAGGTCAAGCCTATTGGACACCATTGGTCGGTTCGCTAAGGCTGGTGCCGATATGTATACCGCTAAGGAACAACGAGCACGAGACCTAGCTGATGAACGCTCTAACGAGATTATCCGTAAGCTGACCCCTGAGCAACGTCGAGAAGCTCTCAACAACGGGACCCTTCTGTATCAGGATGACCCATACGCTATGGAAGCCCTCCGAGTCAAGACTGGTCGTAACGCTGCGTATCTTGTGGCCGATGACGTTATGCAGCAGAt                                                                                                                                                                                                                                                                                                           >  1:38319/1‑301 (MQ=255)`  
        `gggCTATCGTGCAGCAACCACTCAGGCCGAACAGCCAAGGTCAAGCCTATTGGACACCATTGGTCGGTTCGCTAAGGCTGGTGCCGATATGAATACCGCTAAGGAACAACGAGCACGAGACCTAGCTGATGAACGCTCTAACGAGATTATCCGTAAGCTGACCCCTGAGCAACGTCGAGAAGCTCTCAACAACGGGACCCTTCTGTATCAGGATGATCCATACGCTATGGAAGCACTCCGAGTCAAGACTGGTCGTAAAGCTGCGTATCTTGTGGACGATGACGTTATGCAGCAGCTtaaa                                                                                                                                                                                                                                                                                                       >  1:71391/1‑301 (MQ=255)`  
         `ggCTATCGTGCAGCAACCACTCAGGCCGAACAGCCAAGGTCAAGCCTATTGGACACCATTGGTCGGTTCGCTAAGGCTGGTGCCGATATGTATACCGCTAAGGAACAACGAGCACGAGACCTAGCTGATGAACGCTCTAACGAGATTATCCGTAAGCTGACCCCTGAGCAACGTCGAGAAGCTCTCAACAACGGGACCCTTCTGTATCAGGATGACCCATACGCTATGGAAGCACTCCGAGTCAAGACTGGTCGTAACGCTGCGTATCTTGTGGACGATGACGTTATGCAGCAGATAAAag                                                                                                                                                                                                                                                                                                      <  1:207109/301‑1 (MQ=255)`  
         `ggCTATCGTGCAGCAACCACTCAGGCCGAACAGCCAAGGTCAAGCCTATTGGACACCATTGGTCGGTTCGCTAAGGCTGGTGCCGATATGTATACCGCTAAGGAACAACGAGCACGAGACCTAGCTGATGAACGCTCTAACGAGATTATCCGTAAGCTGACCCCTGAGCAACGTCGAGAAGCTCTCAACAACGGGACCCTTCTGTATCAGGATGACCCATACGCTATGGAAGCACTCCGAGTCAAGACTGGTCGTAACGCTGCGTATCTTGTGGACGATGACGTTATGCAGCAGATAAAag                                                                                                                                                                                                                                                                                                      <  1:283426/301‑1 (MQ=255)`  
         `caagaTCGTGCAGCAACCACTCAGGCCGAACAGCCAAGGTCAAGCCTATTGGACACCAGTGGTCGGTTCGCTAAGGCTGGTGCCGATATGTATACCGCTAAGGAACAACGAGCACGAGACCTAGCTGATGAACGCTCTAACGAGATTATCCGTAAGCTGACCCCTGAGCAACGTCGAGAAGGTCTCAACAACGGGACCCTTCTGTATCAGGATGACCCATACGCTATGGAAGCACTCCGAGTCAAGACTGGTCGTAACGCTGCGTATCTTGTGGACGATGACGTTATGCAGCAGATAAAag                                                                                                                                                                                                                                                                                                      <  1:1391/297‑1 (MQ=255)`  
           `cTATCGTGCAGCAACCACTCAGGCCGAACAGCCAAGGTCAAGCCTATTGGACACCATTGGTCGGTTCGCTAAGGCTGGTGCCGATATGTATACCGCTAAGGAACAACGAGCACGAGACCTAGCTGATGAACGCTCTAACGAGATTATCCGTAAGCTGACCCCTGAGCAACGTCGAGAAGCTCTCAACAACGGGACCCTTCTGTATCAGGATGACCCATACGCTATGGAAGCACTCCGAGTCAAGACTGGTCGTAACGCTGCGTATCTTGTGGACGATGACGTTATGCAGCAGATAAAagag                                                                                                                                                                                                                                                                                                    <  1:171795/301‑1 (MQ=255)`  
             `aTCGTGCAGCAACCACTCAGGCCGAACAGCCAAGGTCAAGCCTATTGGACACCATTGGTCGGTTCGCTAAGGCTGGTGCCGATATGTATACCGCTAAGGAACAACGAGCACGAGACCTAGCTGATGAACGCTCTAACGAGATTATCCGTAAGCTGACCCCTGAGCAACGTCGAGAAGCTCTCAACAACGGGACCCTTCTGTATCAGGATGACCCATACGCTATGGAAGCACTCCGAGTCAAGACTGGTCGTAACGCTGCGTATCTTGTGGACGATGACGTTATGCAGCAGATAAAAGAggg                                                                                                                                                                                                                                                                                                  >  1:293623/1‑301 (MQ=255)`  
                         `ccACTCAGGCCGAACAGCCAAGGTCAAGCCTATTAGACACCATTGGTCGGTTCGCTAAGGCTGGTGCCGATATGTATACCGCTAAGGAACAACGAGCACGAGACCTAGCTGATGAACGCTCTAACGAGATTATCCGTAAGCTGACCCCTGAGCAACGTCGAGAAGCTCTCAACAACGGGACCATTCTGTATCAGGATGACCAATACGCTATGGAAGCACTCCGAGTCAAGACTGGTCGTAACGCTGCGTATCTTGTGGACGATGACGTTATGCAGCAGATAAAAGAGGG‑TGTCTTCCGTAc                                                                                                                                                                                                                                                                                     <  1:82947/301‑1 (MQ=255)`  
                          `cACTCAGGCCGAACAGCCAAGGTCAAGCCTATTGGACACCATTGGTCGGTTCGCTAAGGCTGGTGCCGATATGTATACCGCTAAGGAACAACGAGCACGAGACCTAGCTGATGAACGCTCTAACGAGATTATCCGTAAGCTGACCCCTGAGCAACGTCGAGAAGCTCTCAACAACGGGACCCTTCTGTATCAGGATGACCCATACGCTATGGAAGCACTCCGAGTCAAGACTGGTCGTAACGCTGCGTATCTTGTGGACGATGACGTTATGCAGAGGATAACAGAGGG‑TGTCTTCCGTACt                                                                                                                                                                                                                                                                                    >  1:275406/1‑301 (MQ=255)`  
                                    `gAACAGCCAAGGTCAAGCCTATTGGACACCATTGGTCGGTTCGCTAAGGCTGGTGCCGATATGTATACCGCTAAGGAACAACGAGCACGAGACCTAGCTGATGAACGCTCTAACGAGATTATCCGTAAGCTGACCCCTGAGCAACGTCGAGAAGCTCTCAACAACGGGACCCTTCTGTATCAGGATGACCCATACGCTATGGAAGCACTCCGAGTCAAGACTGGTCGTAACGCTGCGTATCTTGTGGACGATTACGTTATGCAGAGGATAAAAGAGGGCTGTCtcttatacacatccccga                                                                                                                                                                                                                                                                           >  1:174136/1‑284 (MQ=255)`  
                                                   `aGCCTATTGGACACCATTGGTCGGTTCGCTAAGGCTGGTGCCGATATGTATACCGCTAAGGAACAACGAGCACGAGACCTAGCTGATGAACGCTCTAACGAGATTATCCGTAAGCTGACCCCTGAGCAACGTCGAGAAGCTCTCAACAACGGGACCCTTCTGTATCAGGATGACCCATACGCTATGGAAGCACTTCGAGTCAAGACTGGTCGTAACGCTGCGTATCTTGTGGACGATGACGTTATGCAGAGGATAAAAGAGGG‑TGTCTTCCGTACTCGCGAAGAGATGGAAGAGTATCGcc                                                                                                                                                                                                                                                           >  1:2040/1‑301 (MQ=255)`  
                                                   `aGCCTATTGGACACCATTGGTCGGTTCGCTAAGGCTGGTGCCGATATGTATACCGCTAAGGAACAACGAGCACGAGACCTAGATGATGAACGCTCTAACGAGATTATCCGTAAGCTGACCCCTGAGCAACGTCGAGAAGCTCTCAACAACGGGACCCTTCTGTATCAGGATGACCCATACGCTATGGAAGCACTCCGAGTCAAGACTGGTCGTAACGCTGCGTATCTTGTGGACGATGACGTTATGCAGCAGATAAAAGAGGG‑TGTCTTCCGTACTCGCGAAGAGATGGAAGAGTAGCGcc                                                                                                                                                                                                                                                           >  1:158816/1‑301 (MQ=255)`  
                                                    `accTATTGGACACCATTGGTCGTTTCGCCAAGGCTGGTGCCGATATGTATACCGCTAAGGAACAACGAGCACGAGACCTAGCTGATGAACGCTCTAACGAGATTATCCGTAAGCTGACCCCTGAGCAACGTCGAGAAGCTCTCAACAACGGGACCCTTCTGGATCAGGATGACCCATACGCTATGGAAGCACTCCGAGTCAAGACTGGTCGTAACGCTGCGTATCTTGTGGGCGATGACGTTATGCAGCAGATAAAAGAGGG‑TGTCTTCCGTACTCGCGAAGAGATGGAAGAGTATCGCCa                                                                                                                                                                                                                                                          <  1:41365/300‑1 (MQ=255)`  
                                                        `ttttgACACCATTGGTCGGTTCGCTAAGGCTGGTGCCGATATGTATACCGCTAAGGAACAACGAGCACGAGACCTAGCTGATGAACGCTCTAACGAGATTATCCGTAAGCTGACCCCTGAGCAACGTCGAGACGCGCTCAACAACGGGACCCTTCTGTATCAGGATGACCCATACGCTATGGAAGCACTCCGAGTCAAGACTTGTCGTAACGCTGCGTATCTTGTGGACGATGACGTTATGCAGAGGATAAAAGAGGG‑TGTCTTCCGTACTCGCGAAGAGATGGAAGAGTATCGCCATAGt                                                                                                                                                                                                                                                      <  1:216791/297‑1 (MQ=255)`  
                                                                `ccATTGGTCGGTTCGCTAAGGCTGGTGCCGATATGTATACCGCTAAGGAACAACGAGCACGAGACCTAGCTGATGAACGCTCTAACGAGATTATCCGTAAGCTGACCCCTGAGCAACGTCGAGAAGCTCTCAACAACGGGACCCTTCTGTATCAGGATGACCCATACGCTATGGAAGCACTCCGAGTCAAGACTGGTCGTAACGCTGCGTATCTTGTGGACGATGACGTTATGCAGCAGATAAAAGAGGG‑TGTCTTCCGTACTCGCGAAGAGATGGAAGAGTATCGCCATAGTCGCCTTCa                                                                                                                                                                                                                                              >  1:237469/1‑301 (MQ=255)`  
                                                                `ccATTGGTCGGTTCGCTAAGGCTGGTGCCGATATGTATACCGCTAAGGAACAACGAGCACGAGACCTAGCTGATGAACGCTCTAACGAGATTATCCGTAAGCTGACCCCTGAGCAACGTCGAGAAGCTCTCAACAACAGGACCCTTCTGTATCAGGATGACCCATACGCTATGGAAGCACTCCGAGTCAAGACTGGTCGTAACGCTGCGTATCTTGTGGACGATGACGTTATGCAGCAGATAAAAGAGGG‑TGTCTTCCGTACTCGCGAAGAGATGGAAGAGTATCGCCactgtctcttata                                                                                                                                                                                                                                              >  1:145211/1‑289 (MQ=255)`  
                                                                             `cGCTAAGGCTGGTGCCGATATGTATACCGCTAAGGAACAACGAGCACGAGACCTAGCTGCTGAACGCTCTAACGAGATTATCCGTAAGCTGACCCCTGCGCAACGTCGAGAAGCTCTCAACAACGGGACCCTTCTGTCTCAGGATGACCCATACGCTATTGAAGCACTCCGAGTCAAGACTGGTCGTAACGCTGCGTATCTTGTGGACGATGATGTCATGCTGTGGTTAACAGCGGG‑TGTCTTCCTTACTCGCGCAGAGATGGAAGAGTATCGCCATCGTCGCTTTCAAGACGGCGCTacg                                                                                                                                                                                                                                 >  1:250050/1‑299 (MQ=255)`  
                                                                             `cGCTAAGGCTGGTGCCGATATGTATACCGCTAAGGAACAACGAGCACGAGACCTAGCTGATGAACGCTCTAGCGAGATTATGCGTAAGCTGACCCCTGAGCAACGTCGAGAAGCTCTCAACAACGGGACCCTTCTGTATCAGGATGACCCATACGCTATGGAAGCACTCCGAGTCAAGACTGGTCGTAACGCTGCGTATCTTGTGGACGATAACGTTATGCAGAAGATAAAAGAGGG‑TGTCTTCCGTACTCGCGAAGAGATGGAAGAGTATCGCCATAGTCGCCTTCAAGAGGGCGCTAAg                                                                                                                                                                                                                                 >  1:302380/1‑301 (MQ=255)`  
                                                                              `gCTAAGGCTGGTGCCGATATGTATACCGCTAAGGAACAACGAGAACGAGACCTAGCTGATGAACGCGCTAACGAGATTATCCGTAAGCTGACCCCTGAGCAACGTCGAGAAGCTCTCAACAACGGGACCCTTCTGTATCAGGATGACCCATACGCTATGGAAGCACTCCGAGTCAAGACTGGTCGTAACGCTGCGTATCTTGTGGACGATGACGTTATGCAGCAGATAAAAGAGGG‑TCTCTTCCGTACTCGCGAAGAGATGGAAGAGTATCGCCATAGTCGCCTTCAAGAGGGCGCTAAgg                                                                                                                                                                                                                                <  1:107313/301‑1 (MQ=255)`  
                                                                                 `aaGGCTGGTGCCGATATGTATACCGCTAAGGAACAACGAGCAAGAGACCTAGCTGATGAACGATCTAACGAGATTATCCGTGAGCTGACCCCTGAGCAACGTCGAGAAGCTCTCAACAACGGGACCCTTCTGTATCAGGATGACCCATACGCTATGGAAGCACTCCGAGTCAAGACTGGTCGTAACGCTGCGTATCTTGTGGACGATGACGTTATGCAGAGGATAAAAGAGGG‑TGTCTTCCGTACTCGCGAAGAGATGGAAGAGTATCGCCATAGTCGCCTTCAAGAGGGCGCTAAGGtat                                                                                                                                                                                                                             <  1:117073/301‑1 (MQ=255)`  
                                                                                   `aaagagacaggATATGTATACCGCTAAGGAACAACGAGCACGAGACCTAGCTGATGAACGCTCTAACGAGATTATCCGTAAGCTGACCCCTGAGCAACGTCGAGAAGCTCTCAACAACGGGACCCTTCTGTATCAGGATGACCCATACGCTATGGAAGCACTCCGAGTCAAGACTGGTCGTAACGCTGCGTATCTTGTGGACGATGACGTTATGCAGAAGATAAAAGAGGG‑TGTCTTCCGTACTCGCGAAGAGATGGAAGAGTATCGCCATAGTCGCCTTCAAGAGGGCGCTAAGGTATAc                                                                                                                                                                                                                           <  1:96813/291‑1 (MQ=255)`  
                                                                                     `taagagacagtatGTATAACGCTAAGGAACAACGAGCACGAGACCTAGCTGATGCACGCTCTAACGAGATTATCCGTAAGCTGACCCCTGAGCAACGTCGAGAAGCTCTCAACAACGGGACCCTTGTGTATCAGGATGACCCATACGGTATGGAAGCACTACGAGTCAAGACTGGTCGTAACGCTGCGTATCTTGTGGACGAAGACGTTATGCAGCAGATAACAGAGGG‑TGTCTTCCGTACTCGCGAAGAAATGGAAGAGTATCGCCATAGTCGCCTTCAAGAGGGCGCTAAGGTATACGc                                                                                                                                                                                                                         <  1:54327/291‑1 (MQ=255)`  
                                                                                     `gtataagagacaggTATACCGCTAAGGAACAACAAGCACGAGACCTAGCTGATGAACGCTCTAACGAGATTATCCGTAAGCTGACCCCTGAGCAACGTCGAGAAGCTCTCAACAACGGGACCCTTCTGTATCAGGATGACCCATACGCTATGGAAGCACTCCGAGTCAAGACTGGTCGTAACGCTGCGTATCTTGTGGACGATGACGTTATGCAGCAGATAAAAGAGGG‑TGTCTTCCGTACTCGCGAAGAGATGGAAGAGTATCGCCATAGTCGCCTTCAAGAGGGCGCTAAGGTATACGc                                                                                                                                                                                                                         <  1:224451/288‑1 (MQ=255)`  
                                                                                     `cttgTGCCGATATTTATACCGCTAAGGAACAACGAGCACGAGACCTAGCTGATGAACGCTCTAACGAGATTATCCGTAAGCTGGCCCCTGAGCAACGTCGAGAAGCTCTCAACAACGGGACCCTTCTGTATCAGGATGACCCATACGCTATGGAAGCACTCCGAGTCAAGACTGGTCGTAACGCTGCGTATCTTGTGGACGATGACGTTATGCAGAAGATAAAAGAGGG‑TGTCTTCCGTACTCGCGAAGAGATGGAAGAGTATCGCCATAGTCGCCTTCAAGAGGGCGCTAAGGTATACGc                                                                                                                                                                                                                         <  1:36428/298‑1 (MQ=255)`  
                                                                                     `cgggTGCCGATATGGATACAGATAAGGAACAACGAGAACGAGACCTAGCTGATGAACGCTCTAAAGAGATTATCCGTAAGCTGACCCCTGAGCAACGTCGAGAAGCTCTCAACAACGGGACCCTTCTGTATCAGGATGACCCATAAGCGATGGAAGCACTCCGAGTCAAGACTGGTCGTAACGCTGCGTATCTTGTGGACGATGACGTTATGCAGCAGATAAAAGAGGG‑TGTCTTCCGTACTCGCGAAGAGATGGAAGAGTATCGCCATAGTCGCCTTCAAGAGGGCGCTAAGGTATACGc                                                                                                                                                                                                                         <  1:307689/299‑1 (MQ=255)`  
                                                                                     `cTGGTGCCGTTATGTAAACCGCTAAGGAACAACGAGCACGAGACCTAGCTGATGAACGCTCTAACGAGATTATCCGTAAGCTGACCCCTGAGCAACGTCGAGAAGCTCTCAACAACGGGACCCTTCTGTATCAGGATGACCCATACGCTATGGAAGCACTCCGAGTCAAGACTTGTCGTAACGCTGCGTATCTTGTGGACGATGACGTTATGCAGCAGATAAAAGAGGG‑TGTCTTCCGTACTCGCGAAGAGATGGAAGAGTATCGCCATAGTCGCCTTCAAGAGGGCGCTAAGGTATACGc                                                                                                                                                                                                                         <  1:329949/301‑1 (MQ=255)`  
                                                                                          `gCCGATATGTATACCGCTAAGGAACAACGAGCACGAGACCTAGCTGATGAACGCTCTAACGAGATTATCCGTAAGCTGACCCCTGAGCAACGTCGAGAAGCTCTCAACAACGGGACCCTTCTGTATCAGGATGACCCATACGCTATGGAAGCACTCCGAGTCAAGACTGGTCGTAACGCTGCGTATCTTGTGGACGATGACGTTATGCAGCAGATAAAAGAGGG‑TGTCTTCCGTACTCGCGAAGAGATGGAAGAGTATCGCCATAGTCGCCTTCAAGAGGGCGCTAAGGTATACGCTGAGc                                                                                                                                                                                                                    >  1:313746/1‑301 (MQ=255)`  
                                                                                            `cagtatGGATACCGCGAAGGAACAACGAGCACGAGACCTAGCTGAGGAACGCTCTAACGAGATGAGCCGTAAGCTGACCCCTGAGCAACGTCGAGAAGCTCTCAACAACGGGACCCTTCTGTAACAGGAGGACCCATACGCTATGGAAGCACTCCGAGTCAAGACTGGTCGTAACGCTGCGTATCTTGTGGACGATGACGTTATGCAGAGGATAAAAGAGGG‑TGTCTTCCGTACTCGCGAAGAGATGGAAGAGTATCGCCATAGTCGCCTTCAAGAGGGCGCTAAGGTATACGCTGAGCAg                                                                                                                                                                                                                  <  1:141390/298‑1 (MQ=255)`  
                                                                                             `gATATGTATACCGCTAAGGAACAACGAGCACGAGACCTAGCTGATGAACGCTCTAACGAGATTATCCGTAAGCTGACCCCTGAGCAACGTCGAGAAGCTCTCAACAACGGGACCCTTCTGTATCAGGATGACCCATACGCTATGGAAGCACTCCGAGTCAAGACTGGTCGTAACGCTGCGTATCTTGTGGACGATGACGTTATGCAGAGGATAAAAGAGGG‑TGTCTTCCGTACTCGCGAAGAGATGGAAGAGTATCGCCATAGTCGCCTTCAAGAGGGCGCTAAggctgtctcttatacac                                                                                                                                                                                                                 >  1:230741/1‑286 (MQ=255)`  
                                                                                             `gATATGTATACCGCTAAGGAACAACGAGCACGAGACCTAGCTGATGAACGCTCTAACGAGATTATCCGTAAGCTGACCCCTGAGCAACGTCGAGAAGCTCTCAACAACGGGACCCTTCTGTATCAGGATGACCCATACGCTATGGAAGCACTCCGAGTCAAGACTGGTCGTAACGCTGCGTATCTTGTGGACGATGACGTTATGCAGAAGATAAAAGAGGG‑TGTCTTCCGTACTCGCGAAGAGATGGAAGAGTATCGCCATAGTCGCCTTCAAGAGGGCGCTAAGGTATACGCTGAGCAGt                                                                                                                                                                                                                 <  1:197774/301‑1 (MQ=255)`  
                                                                                              `atatGTATACCGCTAAGGAACAACGAGCACGAGACCTAGCTGATGAACGCTCTAACGAGATTATCCGTAAGCTGACCCCTGAGCAACGTCGAGAAGCTCTCAACAACGGGACCCTTCTGTATCAGGATGACCCATACGCTATGGAAGCACTCCGAGTCAAGACTGGTCGTAACGCTGCGTATCTTGTGGACGATGACGTTATGCAGCAGATAAAAGAGGG‑TGTCTTCCGTACTCGCGAAGAGATGGAAGAGTATCGCCATAGTCGCCTTCAAGAGGGCGCTAAGGTATACGCTGAGCAGtt                                                                                                                                                                                                                >  1:89408/1‑301 (MQ=255)`  
                                                                                               `tatGTATACCGCTAAGGAACAACGAGCACGAGACCTAGCTGATGAACGCTCTAACGAGATTATCCGTAAGCTGACCCCTGAGCAACGTCGAGAAGCTCTCAACAACGGGACCCTTCTGTATCAGGATGACCCATACGCTATGGAAGCACTCCGAGTCAAGACTGGTCGTAACGCTGCGTATCTTGTGGACGATGACGTTATGCAGCAGATAAAAGAGGG‑TGTCTTCCGTACTCGCGAAGAGATGGAAGAGTATCGCCATAGTCGCCTTCAAGAGGGCGCTAAGGTATACGCTGAGcctgtt                                                                                                                                                                                                               >  1:164166/1‑296 (MQ=255)`  
                                                                                                  `gTATACCGCTAAGGAACAACGAGCACGAGACCTAGCTGATGAACGCTCTAACGAGATTATCCGTAAGCTGACCCCTGAGCAACGTCGAGAAGCTCTCAACAACGGGACCCTTCTGTATCAGGATGACCCATACGCTATGGAATCACTCCGAGTCAAGACTGGTCGTAACGCTGCGTATCTTGTGGACGATGACGTTATGCAGAGGATAAAAGAGGG‑TGTCTTCCGTACTCGCGAAGAGATGGAAGAGTATCGCCATCGTCTCCTTCAAGAGGGCGCTAAGGTATACGCTGAGCAGTTCGGc                                                                                                                                                                                                            >  1:151275/1‑301 (MQ=255)`  
                                                                                                  `gTATACCGCTAAGGAACAACGAGCACGAGACCTAGCTGATGAACGCTCTAACGAGATTATCCGTAAGCTGACCCCTGAGCAACGTCGAGAAGCTCTCAACAACGGGACCCTTCTGTATCAGGATGACCCATACGCTATGGAAACACTCCGAGTCAAGACTGGTCGTAACGCTGCGTATCTTGTGGACGATGACGTTATGCAGAAGATAAAAGAGGG‑TGTCTTCCGTACTCGCGAAGAGATGGAAGAGTATCGCCATAGTCGCCTTCAAGAGGGCGCTAAGGTATACGcctgtctcttatac                                                                                                                                                                                                            >  1:44063/1‑288 (MQ=255)`  
                                                                                                    `ataCCGCTAAGGAACAACGAGCACGAGACCTAGCTGATGAACGCTCTGACGAGATTATCCGTAAGCTGACCCCTGAGCAACGTCGAGAAGCTCTCAACAACGGGACCCTTCTGTATCAGGATGACCCATACGCTATGGTAGCACTCCGAGTCAAGACTGGTCGTAACGCTGCGTATCTTGTGGACGATGACGTTATGCAGAAGATAAAAGAGGG‑TGTCTTCCGTACTCGCGAAGAGATGGAAGAGTATCGCCATAGTCGCCTTCAAGAGGGCGCTAAGGTAAACGCTGAGCAGTTCGGCAt                                                                                                                                                                                                          >  1:346979/1‑301 (MQ=255)`  
                                                                                                    `ataCCGCTAAGGAACAACGAGCACGAGACCTAGCTGATGAACGCTCTAACGAGATTATCCGTAAGCTGACCCCTGAGCAACGTCGAGAAGCTCTCAACAACGGGACCCTTCTGTATCAGGATGACCCATACGCTATGGAAGCACTCCGAGTCAAGACTGGTCGTAACGCTGCGTATCTTGTGGACGATGACGTTATGCAGAGGATAAAAGAGGG‑TGTCTTCCGTACTCGCGAAGAGATGGAAGAGTATCTCCATAGTCGCCTTCAAGAGGGCGCTAAGGTATACGCTGAGCAGTTCGGCAt                                                                                                                                                                                                          <  1:76285/301‑1 (MQ=255)`  
                                                                                                          `cTAAGGAACAACGAGCACGAGACCTAGCTGATGAACGCTCTAACGAGATTATCCGTAAGCTGACCCCTGAGCAACGTCGAGAAGCTCTCAACAACGGGACCCTTCTGTATCAGGATGACCCATACGCTATGGAAGCACTCCGAGTCAAGACTGGTCGTAACGCTGCGTATCTTGTGGACGATGACGTTATGCAGCAGATAAAAGAGGG‑TGTCTTCCGTACTCGCGACGAGATGGAAGAGTATCGCCATAGTCGCCTTCAAGAGGGCGCTAAGGTATACGCTGAGCAGTTCGGCATCGAccc                                                                                                                                                                                                    >  1:129794/1‑301 (MQ=255)`  
                                                                                                            `aaGGAACAACGAGCACGAGACCTAGCTGATGAACGCTCTAACGAGATTATCCGTAAGCTGACCCCTGAGCAACGTCGAGAAGCTCTCAACAACGGGACCCTTCTGTATCAGGATGACCCATACGCTATGGAAGCACTCCGAGTCAAGACTGGTCGTAACGCTGCGTATCTTGTGGACGATGACGTTATGCAGAGGATAAAAGAGGG‑TGTCTTCCGTACTCGCGAAGAGATGGAAGAGTATCGCCATAGTCGCCTTCAAGAGGGCGCTAAGGTATACGCTGAGcctgtctcttatacacatc                                                                                                                                                                                                  >  1:254301/1‑283 (MQ=255)`  
                                                                                                              `ggAACAACGAGCACGAGACCTAGCTGATGAACGCTCTAACGAGATTATCCGTAAGCTGACCCCTGAGCAACGTCGAGAAGCTCTCAACAACGGGACCCTTCTGTATCAGGATGACCCATACGCTATGGAAGCACTCCGAGTCAAGACTGGTCGTAACGCTGCGTATCTTGTGGACGATGACGTTATGCAGCAGATAAAAGAGGG‑TGTCTTCCGTACTCGCGAAGAGATGGAATAGTATCGCCATAGTCGCCTTCAAGAGGGCGCTAAGGTATACGCTGAGCAGTTCGGCCTCGACCCTGAg                                                                                                                                                                                                >  1:287304/1‑301 (MQ=255)`  
                                                                                                                `tgtgtataagagccagccTAGCTGATGAACGCTCTAACGAGATTATCCGTAAGCTGACCCCTGAGCAACGTCGAGAAGCTCTCAACAACGGGACCCTTCTGTATCAGGATGACCCATACGCTATGGAAGCACTCCGAGTCAAGACTGGTCGTAACGCTGCGTATCTTGTGGACGATGACGTTATGCAGCAGATAAAAGAGGG‑TGTCTTCCGTACTCGCGAAGAGATGGAAGAGTATCGCCATAGTCGCCTTCAAGAGGGCGCTAAGGTATACGCTGAGCAGTTCGGCATCGACCCTGAGGa                                                                                                                                                                                              <  1:296842/285‑1 (MQ=255)`  
                                                                                                                  `aaacGAGCACGAGACCTAGCTGATGAACGCTCTAACGAGATTATCCGTAAGCTGACCCCTGAGCAACGTCGAGAAGCTCTCAACAACGGGACCCTTCTGTATCAGGATGACCCATACGCTATGGAAGCACTCCGAGTCAAGACTGGTTGTAACGCTGCGTATCTTGTGGACGATGACGTTATGCAGAGGATAAAAGAGGG‑TGTCTTCCGTACTCGCGAAGAGATGGAAGAGTATCGCCATAGTCGCCTTCAAGAGGGCGCTAAGGTATACGCTGAGCAGTTCGGCATCGACCCTGAGGACg                                                                                                                                                                                            <  1:290078/300‑1 (MQ=255)`  
                                                                                                                         `caagagaCCTAGCGGATGAACGCTCTAACGAGATTATCCGTAAGCTGACCCCTGAGCAACGTCGAGAAGCTCTCAACAACGGGACCCTTCTGTATCAGGATGACCCATACGCTATGGAAGCACTCCGAGTCAAGACTGGTCGTAACGCTGCGTATCTTGTGGACGATGACGTTATGCAGCAGATAAAAGAGGG‑TGTCTTCCGTACTCGCGAAGAGATGGAAGAGTATCGCCATAGTCGCCTTCAAGAGGGCGCTAAGGTATACGCTGAGCAGTTCGGCATCGACCCTGAGGACGTTGATTa                                                                                                                                                                                     <  1:214967/298‑1 (MQ=255)`  
                                                                                                                           `cGAGACCTAGCTGATGAACGCTCTAACGAGATTATCCGTAAGCTGACCCCTGAGCAACGTCGAGAAGCTCTCAACAACGGGACCCGTCTGTATCAGGATGACCCATACGCTATGGAAGCACTCCGAGTCAAGACTGGTCGTAACGCTGCGTATCTTGTGGACGATGACGTTATGCAGAGGATAAAAGAGGG‑TGTCTTCCGTACTCGCGAAGAGATGGAAGAGTATCGCCATAGTCGCCTTCAAGAGGGCGCTAAGGTATACGCTGAGCAGTTCGGCATCGACCCTGAGGACGTTGATTAtg                                                                                                                                                                                   <  1:132577/301‑2 (MQ=255)`  
                                                                                                                             `ttgtgtctaagcgccaggCTCTCACGAGATTATCCGTAAGCTGACCCCTGAGCAACGTCGAGAAGCTCTCAACAACGGGACCCTTCTGTATCAGGATGACCCATACGCTATGGAAGCACTCCGAGTCAAGACTGGTCGTCACGCTGCGTATCTTGTGGACGATGACGTTATTCAGAAGATAAAAGAGGG‑TGTCTTCCGTACTCGCGAAGAGATGGAAGAGTATCGCCATAGTCGCCTTCAAGAGGGCGCTAAGGTATACGCTGAGCAGTTCGGCATCGACCCTGAGGACGTTGATTATCAg                                                                                                                                                                                 <  1:193269/284‑1 (MQ=255)`  
                                                                                                                                  `tAGCTGATGAACGCTCTAACGAGATTATCCATAAGCTGACCCCTGAGCAACGTCGAGAAGCTCTCAACAACGGGACCCTTCTGTATCAGGATGACCCATACGCTATGGAAGCACTCCGAGTCAAGACTGGTCGTAACGCTGCGTATCTTGTGGACGATGACGTTATGCAGCAGATAAAAGAGGG‑TGTCTTCCGTACTCGCGAAGAGATGGAAGAGTATCGCCATAGTCGCCTTCAAGAGGGCGCTAAGGTATACGCTGAGCAGTTCGGCATCGACCCTGAGGACGTTGATTATCAGCGTgg                                                                                                                                                                            >  1:260918/1‑301 (MQ=255)`  
                                                                                                                                             `ggggctcggagatgtgtataagagacagccccTGAGCAACGTCGAGAAGCTCTCAACAACGGGACCCTTCTGTAACAGGATGACCCATACGCTATGGAAGCACTCCGAGTCAAGACTGGTCGTAACGCTGCGTATCTTGTGGACGATGACGTTATGCAGAAGATAAAAGA‑GG‑TGTCTTCCGTACTCGCGAAGAGATGGAAGAGTATCGCCATAGTCGCCTTCAAGAGGGCGCTAAGGTATACGCTGAGCAGTTCGGCATCGACCCTGAGGACGTTGATTATCAGCGTGGTTTCAACGGGGa                                                                                                                                                                <  1:94947/273‑1 (MQ=255)`  
                                                                                                                                               `ctctAACGAGATTATCCGTAAGCTGACCCCTGAGCAACGTCGAGAAGCTCTCAACAACGGGACCCTTCTGTATCAGGATGACCGATACGCTATTGAAGCACTCCGAGTCAAGACTGGTCGTAACGCTGCGTATCTTGTGGACGATGACGTTATGCAGCAGATAAAAGAGGG‑TGTCTTCCGTACTCGCGAAGAGATGGAAGAGTATCGCCATAGTCGCCTTCAAGAGGGCGCTAAGGTATACGCTGAGCAGTTCGGCATCGACCCTGAGGACGTTGATTATCAGCGTGGTTTCAACGAGGAc                                                                                                                                                               <  1:170038/301‑1 (MQ=255)`  
                                                                                                                                                 `ctAACGAGATTATCCGTAAGCTGACCCCTGAGCAACGTCGAGAAGCTCTCAACAACGGGACCCTTCTGTATCAGGATGACCCATACGCTATGGAAGCACTCCGAGTCAAGACTGGTCGTAACGCTGCGTATCTTGTGGACGATGACGTTATGCAGAAGATAAAAGAGGG‑TGTCTTCCGTACTCGCGAAGAGATGGAAGAGTATCGCCATAGTCGCCTTCAAGAGGGCGCTAAGGTATACGCTGAGCAGTTCGGCATCGACCCTGAGGACGTTGATTATCAGCGTGGTTTCAACGGGGACAt                                                                                                                                                             >  1:114099/1‑301 (MQ=255)`  
                                                                                                                                                        `gaTTATCCGTAAGCTGACCCCTGAGCAACGTCGAGAAGCTCTCAACAACGGGACCCTTCTGTATCAGGATGACCCATACGCTATGGAAGCACTCCGAGTCAAGACTGGTCGTAACGCTGCGTATCTTGTGGACGATGACGTTATGCAGAGGATAAAAGAGGG‑TGTCTTCCGTACTCGCGAAGAGATGGAAGAGTATCGCCATAGTCGCTTTCAAGAGGGCGCTAAGGTATACGCTGAGCAGTTCGGCATCGACCCTGAGGACGTTGATTATCAGCGTGGTTTCAACGGGGACATTACCGAg                                                                                                                                                      >  1:161553/1‑301 (MQ=255)`  
                                                                                                                                                         `aTTAGCCGTAAGCTGACGCGTGAGCAACGACGAGAAGCTCTCAACAACGGGACGCTGCTGTATCAGGATGACCCAGACGCTATGGAAGCACTCCGAGTCAAGAGTGGTCGTAACGCTGCGGATCTTGTGGAAGATGACGTTATGCAGAAGATAAAAGAGGG‑TGTCTTCCGTAATCGCGAAGAGATGGAAGAGTATCGCCATAGTCGCCTTAAAGAGGGCGCTAAGGTATACGGTGAGCAGTTCGGCATCGACCCTGAGGACGTTGATTATCAGCGTGGTTTCAACGGGGACATTACCGAGc                                                                                                                                                     <  1:345054/301‑1 (MQ=255)`  
                                                                                                                                                                    `gCTGACCCCTGAGCAACGTCGAGAAGCTCTCAACAACGGGACCCTTCTGTATCAGGATGACCCATACGCTATGGAAGCACTTCGAGTCAAGACTGGTCGTAACGCTGCGTATCTTGTGGACGATGACGTTATGCAGCAGATAAAAGAGGG‑TGTCTTCCGTACTCGCGAATAGATGGAAGAGTACAGCCATAGTCGCCTTCAAGGGGCCGCCAAGGTTTACGCTGAGCAGTTCGGCATCGACCCTGAGCACGTTGATTATCCGCGTGGTTTCAACGGGGGCATTACCGAGCGTAAccatttg                                                                                                                                          >  1:324364/1‑295 (MQ=255)`  
                                                                                                                                                                       `gACCCCTGAGCAACGTCGAGAAGCTCTCAACAACGGGACCCTTCTGTATCAGGATGACCCATACGCTATGGAAGCACTCCGAGTCAAGACTGGTCGTAACGCTGCGTACCTTGTGGACGATGACGTTATGCAGCAGATAAAAGAGGG‑TGTCTTCCGTACTCGCGAAGAGATGGAAGAGTATCGCCATAGTCGCCTTCAAGAGGGCGCTAAGGTATACGCTGAGCAGTTCGGCATCGACCCTGAGGACGTTGATTATCAGCGTGGTTTCAACGGGGACATTACCGAGCGTAACATCTCGCTg                                                                                                                                       >  1:26964/1‑301 (MQ=255)`  
                                                                                                                                                                       `cggagatgtgtataagagacaggCTCTCAACAACGGGACCCTTCTGTATCAGGATGACCCATACGCTATGGAAGCACTCCGAGTCAAGACTGGTCGTAACGCTGCGTATCTTGTGGACGATGACGTTATGCAGAAGATAAAAGAGGG‑TGTCTTCCGTACTCGCGAAGAGATGGAAGAGTATCGCCATAGTCGCCTTCAAGAGGGCGCTAAGGTATACGCTGAGCAGTTCGGCATCGACCCTGAGGACGTTGATTATCAGCGTGGTTTCAACGGGGACATTACCGAGCGTAACATCTCGCTg                                                                                                                                       <  1:257513/279‑1 (MQ=255)`  
                                                                                                                                                                          `cccTGAGCAACGTCGAGAAGCTCTCAACAACGGGACCCTTCTGTATCAGGATGACCCATACGCTATGGAAGCACTCCGAGTCAAGACTGGTCGTAACGCTGCGTATCTTGTGGACGATGACGTTATGCAGAAGATAAAAGAGGG‑TGTCTTCCGTACTCGCGAAGAGATGGAAGAGTATCGCCATAGTCGCCTTCAAGAGGGCGCTAAGGTATACGCTGAGCAGTTCGGCATCGACCCTGAGGACGTTGATTATCAGCGTGGTTTCAACGGGGACATTACCGAGCGTAACATCTCGCTGTAt                                                                                                                                    >  1:186260/1‑301 (MQ=255)`  
                                                                                                                                                                                     `gTCGAGAAGCTCTCAACAACGGGACCCTTCTGTATCAGGATGACCCATACGCTATGGAAGCACTCCGAGTCAAGACTGGTCGTAACGCTGCGTATCTTGTGGACGATGACGTTATGCAGAGGATAAAAGAGGG‑TGTCTTCCGTACTCGCGAAGAGATGGAAGAGTATCGCCATAGTCGCCTTCAAGAGGGCGCTAAGGTATACGCTGAGCAGTTCGGCATCGACCCTGAGGACGTTGATTATCAGCGTGGTTTCAACGGGGACATTACCGAGCGTAACATCTCGCTGTCTgtctcttatac                                                                                                                         >  1:272675/1‑291 (MQ=255)`  
                                                                                                                                                                                               `cctcAACAACGGGACCCTTCTGTATCAGGATGACCCATACGCTATGGAAGCACTCCGAGTCAAGACTGGTCGTAACGCTGCGTATCTTGTGGACGATGACGTTATGCAGAAGATAAAAGAGGG‑TGTCTTCCGTACTCGCGAAGAGATGGAAGAGTATCGCCATAGTCGCCTTCAAGAGGGCGCTAAGGTATACGCTGAGCAGTTCGGCATCGACCCTGAGGACGTTGATTATCAGCGTGGTTTCAACGGGGACATTACCGAGCGTAACATCTCGCTGTATGGTGCGCAAGATAACTTCTTg                                                                                                               <  1:148248/300‑1 (MQ=255)`  
                                                                                                                                                                                                  `caacaaCGGGACCCTTCTGTATCAGGATGACCCATACGCTATGGAAGCACTCCGAGTCAAGACTGGTCGTAACGCTGCGTATCTTGTGGACGATGACGTTATGCAGCAGATAAAAGAGGG‑TGTCTTCCGTACTCGCGAAGAGATGGAAGAGTATCGCCATAGTCGCCTTCAAGAGGGCGCTAAGGTATACGCTGAGCAGTTCGGCATCGACCCTGAGGACGTTGATTATCAGCCTGGTTTCAACGGGGACATTACCGAGCGTAACATCTCGCTGTATGGTgctgtctcttatacacatctc                                                                                                            >  1:190621/1‑282 (MQ=255)`  
                                                                                                                                                                                                     `caacGGGACCCTTCTGTATCAGGATGACCCATACGCTATGGAAGCACTCCGAGTCAAGACTGGTCGTAACGCTGCGTATCTTGTGGACGATGACGTTATGCAGAGGATAAAAGAGGG‑TGTCTTCCGTACTCGCGAAGAGATGGAAGAGTATCGCCATAGTCGCCTTCAAGAGGGCGCTAAGGTATACGCTGAGCAGTTCGGCATCGACCCTGAGGACGTTGATTATCAGCGTGGTTTCAACGGGGACATTACCGAGCGTAACATCTCGCTGTATGGTGCGCATGATAActtctgtctcttt                                                                                                         >  1:12530/1‑293 (MQ=255)`  
                                                                                                                                                                                                             `cccTTCTGTATCAGGATGACCCATACGCTATGGAAGCACTCCGAGTCAAGACTGATCGTAACGCTGCGTATCTTTTGGACGATTACGTTATGCAGCAGATAAAAGAGGG‑TGTCTTCCGTACTCGCGCAGCGTTGGAAGAGTATCGCCATAGTCGCCTTCCAGAGGGCGCTACGGTCTACGCTGACCAGTTCAGCATCGACCCTGAGGACGTTGATTATCACCGTGGTTTCAACGGGGCCATTACCGAGCTTAACTGCTCCCTGTATGGTGGGCATGATAACTTCTTGACTCAGCAAGcccc                                                                                                 >  1:338687/1‑298 (MQ=255)`  
                                                                                                                                                                                                                `ttCTGTATCAGGATGACCCATACGCTATGGAAGCACTCCGAGTCAAGACTGGTCGTAACGCTGCGTATCTTGTGGACGATGACGTTATGCAGAGGATAAAAGAGGG‑TGTCTTCCGTACTCGCGAAGAGATGGAAGAGTATCGCCATAGTCGCCTTCAAGAGGGCGCTAAGGTATACGCTGAGCAGTTCGGCATCGACCCTGAGGACGTTGATTATCAGCGTGGTTTCAACGGGGACATTACCGAGCGTAACATCTCGCTGTATGGTGCGCATGATAACTTCTTGAGCCAGCAAGCTCAGaa                                                                                              >  1:310020/1‑301 (MQ=255)`  
                                                                                                                                                                                                                 `tCTGCATCAGGATGACCCATACGCTATGGAAGCACTCCGAGTCAAGACTGATCGTAACGCTGCGTATCTTGTGGACGATGACGTTATGCAGAAGATAAAAGAGGG‑TGTCTTCCGTACTCGCGAAGAGATGGAAGAGTATCGCCATAGTCGCCTTCAAGAGGGCGCTAAGGTATACGCTGAGCAGTTCGGCATCGACCCTGAGGACGTTGATTATCAGCGTGGTTTCAACGGGGACATTACCGAGCGTAACATCTCGCTGTATGGTGCGCATGATAACTTCTTGAGCCAGCAAGCTCAGAAg                                                                                             <  1:154519/301‑1 (MQ=255)`  
                                                                                                                                                                                                                    `gTATCAGGATGACCCATACGCTATGGAAGCACTCCGAGTCAAGACTGGTCGTAACGCTGCGTATCTTGTGGACGATGACGTTATGCAGAAGATAAAAGAGGG‑TGTCTTCCGTACTCGCGAAGAGATGGAAGAGTATCGCCATAGTCGTCTTCAAGAGGGCGCTAAGGTATACGCTGAGCAGTTCGGCATCGACCCTGAGGACGTTGATTATCAGCGTGGTTTCAACGGGGACATTACCGAGCGTAACATCTCGCTGTATGGTGCGCATGATAACTTCTTGAGCCAGCAAGCTCAGAAGGgc                                                                                          >  1:246487/1‑301 (MQ=255)`  
                                                                                                                                                                                                                     `aaatgggtataagagacagCTATGGAAGCACGCCGAGTCAAGACGGGTCGTAACGCTGCGTATCTTGTGGACGATGACGTTATGCAGATGATAAAAGAGGG‑TGTCTTCCGTACTCGCGAAGAGATGGAAGAGTATCGCCATAGTCGCCTTCAAGAGGGCGCTAAGGTATACGCTGAGCAGTTCGGCATCGACCCTGAGGACGTTGATTATCAGCGTGGTTTCAACGGGGACATTACCGAGCGTAAAATCTCGCTGTATGGTGCGCATGATAACTTCTTGAGCCAGCAAGCTCAGAAGGgcg                                                                                         <  1:30002/283‑1 (MQ=255)`  
                                                                                                                                                                                                                          `ggATGACCCATACGCTATGGAAGCACTCCGAGTCAAGACTGGTCGTAACGCTGCGTATCTTGTGGACGATGACGTTATGCAGAGGATAAAAGAGGG‑TGTCTTCCGTACTCGCGAAGAGATGGAAGAGTATCGCCATAGTCGCCTTCAAGAGGGCGCTAAGGTATACGCTGAGCAGTTCGGCATCGACCCTGAGGACGTTGATTATCAGCGTGGTTTCAACGGGGACATTACCGAGCGTAACATCTCGCTGTATGGTGCGCATGATAACTTCTTGAGCCAGCAAGCTCAGAAGGGCGCTATc                                                                                    >  1:86179/1‑301 (MQ=255)`  
                                                                                                                                                                                                                              `gACCCATACGCTATGGAAGCACTCCGAGTCAAGACTGGTCGTAACGCTGCGTATCTTGTGGACGATGACGTTATGCAGCAGATAAAAGAGGG‑TGTCTTCCGTACTCGCGAAGAGATGGAAGAGTATCGCCATAGTCGCCTTCAAGAGGGCGCTAAGGTATACGCTGAGCAGTTCGGCATCGACCCTGAGGACGTTGATTATCAGCGTGGTTTCAACGGGGACATTACCGAGCGTAACATCTCGCTGTATGGTGCGCATGATAACTTCTTGAGCCAGCAAGCTCAGAAGGGCGCTATCATGa                                                                                >  1:57174/1‑301 (MQ=255)`  
                                                                                                                                                                                                                                `ccctttcGCTATGAACGCACTCCGAGTCAAGACTGGTCGTAACGCTGCGTATCTTGTGGACGATGACGTTATGCAGAGGATAAAAGAGGG‑TGTCTTCCGTACTCGCGAAGAGATGGAAGAGTATCGCCATAGTCGCCTTCAAGAGGGCGCTAAGGGATACGCTGAGCAGTTCGGCATCGACCCTGAGGACGTTGATTATCAGCGTGGTTTCAACGGGGACATTACCGAGCGTAACATCTCGCTGTATGGTGCGCATGATAACTTCTTGAGCCAGCAAGCTCAGAAGGGCGCTATCATGAAc                                                                              <  1:74190/295‑1 (MQ=255)`  
                                                                                                                                                                                                                                `cccATACGCTATGGAAGCACTCCGAGTCAAGACTGGTCGTAACGCTGCGTATCTTGTGGACGATGACGTTATGCAGCAGATAAAAGAGGG‑TGTCTTCCGTACTCGCGAAGAGATGGAAGAGTATCGCCATAGTCGCCTTCAAGAGGGCGCTAAGGTATACGCTGAGCAGTTCGGCATCGACCCTGAGGACGTTGATTATCAGCGTGGTTTCAACGGGGACATTACCGAGCGTAACATCTCGCTGTATGGTGCGCATGATAACTTCTTGAGCCAGCAAGCTCAGAAGGGCGCTATCATGAAc                                                                              >  1:79366/1‑301 (MQ=255)`  
                                                                                                                                                                                                                                `cccATACGCTATGGAAGCACTCCGAGTCAAGACTGGTCGTAACGCTGCGTATCTTGTGGACGATGACGTTATGCAGAGGATAAAAGAGGG‑TGTCTTCCGTACTCGCGAAGAGATGGAAGAGTATCGCCATAGTCGCCTTCAAGAGGGCGCTAAGGTATACGCTGAGCAGTTCGGCATCGACCCTGAGGACGTTGATTATCAGCGTGGTTTCAACGGGGACATTACCGAGCGTAACATCTCGCTGTATGGTGCGCATGATAACTTCTTGAGCCAGCAAGCGCAGAAGGGCGCTATCATGaaa                                                                              >  1:209246/1‑300 (MQ=255)`  
                                                                                                                                                                                                                                `cccATACGCTATGGAAGCACTCCGAGTCAAGACTGGTCGTAACGCTGCGTATCTTGTGGACGATGACGTTATGCAGAAGATAAAAGAGGG‑TGTCTTCCGTACTCGCGAAGAGATGGAAGAGTATCGCCATAGTCGCCTTCAAGAGGGCGCTAAGGTATACGCTGAGCAGTTCGGCATCGACCCTGAGGACGTTGATTATCAGCGTGGTTTCAACGGGGACATTACCGAGCGTAACATCTCGCTGTATGGTGCGCATGATAACTTCTTGAGCCAGCAAGCTAAGAAGGGCGCTATCCTGAAc                                                                              <  1:305035/301‑1 (MQ=255)`  
                                                                                                                                                                                                                                   `attcGCTAAGGAAGAACTCCGGATCAAGTCTGGTCGTAACGCTTCGTATCTTATGGACGATGACTTTATGCAGAGGATAAAAGAGGG‑TGTCTTCCGTACGCGCGAAGAGATTGAAGAGTATCGCCATAGTCGCCTTAAAGAAGGCGCTAAGGTATACGCTGAGCAGTTCGGCATCGACCCTGAGGACGTTGATTATCAGCGTGGTTTCAACGGGAACATTACCGAGCGTAACATATCGATGTATGGTGCGCATGATAACTTCTTGAGCCAGCAAGCTCAGAAGGGCGCTATCATGAACAGc                                                                           <  1:60411/298‑1 (MQ=255)`  
                                                                                                                                                                                                                                        `cTATGGAAGCACTCCGAGTCAAGACTGGTCGTAACGCTGCGTATCTTGTGGACGATGACGTTATGCAGCAGATAAAAGAGGG‑TGTCTTCCGTACTCGCGAAGAGATGGAAGAGTATCGCCATAGTCGCCTTCAAGAGGGCGCTAAGGTATACGCTGAGCAGTTCGGCATCGACCCTGAGGACGTTGATTATCAGCGTGGTTTCAACGGGGACATTACCGAGCGTAACATCTCGCTGTATGGTGCGCATGATAACTTCTTGAGCCAGCAAGCTCAGAAGGGCGCTATCATGAACAGCCGAGt                                                                      >  1:320533/1‑301 (MQ=255)`  
                                                                                                                                                                                                                                        `cTATGGAAGCACTCCGAGTCAAGACTGGTCGTAACGCTGCGTATCTTGTGGACGATGACGTTATGCAGCAGATAAAAGAGGG‑TGTCTTCCGTACTCGCGAAGAGATGGAAGAGTATCGCCACAGTCGCCTTCAAGAGGGCGCTAAGGTATACGCTGAGCAGTTCGGCATCGACCCTGAGGACGTTGATTATCAGCGTGGTTTCAACGGGGACATTACCGAGCGTAACATCTCGCTGTATGGTGCGCATGATAACTTCTTGAGCCAGCAAGCTCAGAAGGGCGCTATCATGAACAGcctgtc                                                                      >  1:2004/1‑297 (MQ=255)`  
                                                                                                                                                                                                                                        `cTATGGAAGCACTCCGAGTCAAGACTGGTCGTAACGCTGCGTATCTTGTGGACGATGACGTTATGCAGAGGATAAAAGAGGG‑TGTCTTCCGTACTCGCGAAGAGATGGAAGAGTATCGCCATAGTCGCCTTCAAGAGGGCGCTAAGGTATACGCTGAGCAGTTCGGCATCGACCCTGAGGACGTTGATTATCAGCGTGGTTTCAACGGGGACATTACCGAGCGTAACATCTCGCTGTATGGTGCGCATGATAACTTCTTGAGCCAGCAAGCTCAGAAGGGCGCTATCATGAACAGCCGAGt                                                                      >  1:279329/1‑301 (MQ=255)`  
                                                                                                                                                                                                                                          `aTGGAAGCACTCCGAGTCAAGACTGGTCGTAACGCTGCGTATCTTGTGGACGATGACGTTATGCAGAGGATAAAAGAGGG‑TGTCTTCCGTACTCGCGAAGAGATGGAAGAGTATCGCCATAGTCGCCTTCAAGAGGGCGCTAAGGTATACGCTGAGCAGTTCGGCATCGACCCTGAGGACGTTGATTATCAGCGTGGTTTCAACGGGGACATTACCGAGCGTAACATCTCGCTGTATGGTGCGCATGATAACTTCTTGAGCCAGCAAGCTCAGAAGGGCGCTATCATGAACAGCCGAGTgg                                                                    >  1:252123/1‑301 (MQ=255)`  
                                                                                                                                                                                                                                                   `cTCCGAGTCAAGACTGGTCGTAACGCTGCGTATCTTGTGGACGATGACGTTATGCAGAGGATAAAAGAGGG‑TGTCTTCCGTACTCGCGAAGAGATGGAAGAGTATCGCCATAGTCGCCTTCAAGAGGGCGCTAAGGTATACGCTGAGCAGTTCGGCATCGACCCTGAGGACGTTGATTATCAGCGTGGTTTCAACGGGGACATTACCGAGCGTAACATCTCGCTGTATGGTGCGCATGATAACTTCTTGAGCCAGCAAGCTCAGAAGGGCGCTATCCTGAACAGCCTAGTGGAACTCAACg                                                           >  1:180393/1‑301 (MQ=255)`  
                                                                                                                                                                                                                                                       `gAGTCAAGACTGGTCGTAACGCTGCGTATCTTGTGGACGATGACGTTATGCAGAAGATAAAAGAGGG‑TGTCTTCCGTACTCGCGAAGAGATGGAAGAGTATCGCCATAGTCGCCTTCAAGAGGGCGCTAAGGTATACGCTGAGCAGTTCGGCATCGACCCTGAGGACGTTGATTATCAGCGTGGTTTCAACGGGGACATTACCGAGCGTAACATCTCGCTGTATGGTGCGCATGATAACTTCTTGAGCCAGCAAGCTCAGAAGGGCGCTATCATGAACAGCCGAGTGGAACTCAACGgtgt                                                       >  1:263580/1‑301 (MQ=255)`  
                                                                                                                                                                                                                                                             `aGACTGGTCGTAACGCTGCGTATCTTGTGGACGATGACGTTATGCAGAGGATAAAAGAGGG‑TGTCTTCCGTACTCGCGAAGAGATGGAAGAGTATCGCCATAGTCGCCTTCAAGAGGGCGCTAAGGTATACGCTGAGCAGTTCGGCATCGACCCTGAGGACGTTGATTATCAGCGTGGTTTCAACGGGGACATTACCGAGCGTAACATCTCGCTGTATGGTGCGCATGATAACTTCTTGAGCCAGCAAGCTCAGAAGGGCGCTATCATGAACAGCCGAGTGGCACTCAACGGTGGCCTTcc                                                 >  1:317061/1‑300 (MQ=255)`  
                                                                                                                                                                                                                                                                 `tgtataagggacagCGTATCTTGTGGACGATGACGTTATGCAGAGGATAAAAGAGGG‑TGTCTTCCGTACTCGCGAAGAGATGGAAGAGTATCGCCATAGTCGCCTTCAAGAGGGCGCTAAGGTATACGCTGAGCAGTTCGGCATCGACCCTGAGGACGTTGATTATCAGCGTGGTTTCAACGGGGACATTACCGAGCGTAACATCTCGCTGTATGGTGCGCATGATAACTTCTTGAGCCAGCAAGCTCAGAAGGGCGCTATCATGAACAGCCGAGTGGAACTCAACGGTGTCCTTCAAGAc                                             <  1:167278/288‑1 (MQ=255)`  
                                                                                                                                                                                                                                                                 `tGGTCGTAACGCTGCGTATCTTGTGGACGATGACGTTATGCAGAAGATAAAAGAGGG‑TGTCTTCCGTACTCGCGAAGAGATGGAAGAGTATCGCCATAGTCGCCTTCAAGAGGGCGCTAAGGTATACGCTGAGCAGTTCGGCATCGACCCTGAGGACGTTGATTATCAGCGTGGTTTCAACGGGGACATTACCGAGCGTAACATCTCGCTGTATGGTGCGCATGATAACTTCTTGAGCCAGCAAGCTCAGAAGGGCGCTATCATGAACAGCCGAGTGGAACTCAACGGTGTCCTTCAAGAc                                             <  1:136417/301‑1 (MQ=255)`  
                                                                                                                                                                                                                                                                 `gggggatcggagatgtgtataagagacagATGACGTTATGCAGAAGATAAAAGAGGG‑TGTCTTCCGTACTCGCGAAGAGATGGAAGAGTATCGCCATAGTCGCCTTCAAGAGGGCGCTAAGGTATACGCTGAGCAGTTCGGCATCGACCCTGAGGACGTTGATTATCAGCGTGGTTTCAACGGGGACATTACCGAGCGTAACATCTCGCTGTATGGTGCGCATGATAACTTCTTGAGCCAGCAAGCTCAGAAGGGCGCTATCATGAACAGCCGAGTGGAACTCAACGGTGTCCTTCAAGAc                                             <  1:21180/273‑1 (MQ=255)`  
                                                                                                                                                                                                                                                                 `agatgtatataagagacagcTTGTGGACGATGACGTTATGCAGCAGATAAAAGAGGG‑TGTCTTCCGTACTCGCGAAGAGATGGAAGAGTATCGCCATAGTCGCCTTCAAGAGGGCGCTAAGGTATACGCTGAGCAGTTCGGCATCGACCCTGAGGACGTTGATTATCAGCGTGGTTTCAACGGGGACATTACCGAGCGTAACATCTCGCTGTATGGTGCGCATGATAACTTCTTGAGCCAGCAAGCTCAGAAGGGCGCTATCATGAACAGCCGAGTGGAACTCAACGGTGTCCTTCAAGAc                                             <  1:26568/282‑1 (MQ=255)`  
                                                                                                                                                                                                                                                                  `ggTCGTAACGCTGCGTATCTTGTGGACGATGACGTTATGCAGAGGATAAAAGAGGG‑TGTCTTCCGTACTCGCGAAGAGATGGAAGAGTATCGCCATAGTCGCCTTCAAGAGGGCGCTAAGGTATACGCTGAGCAGTTCGGCATCGACCCTGAGGACGTTGATTATCAGCGTGGTTTCAACGGGGACATTACCGAGCGTAACATCTCGCTGTATGGTGCGCATGATAACTTCTTGAGCCAGCAAGCTCAGAAGGGCGCTATCATGAACAGCCGAGTGGAACTCAACGGTGCCCCTCAAGAcc                                            >  1:221074/1‑301 (MQ=255)`  
                                                                                                                                                                                                                                                                   `gTCGTAACGCTGCGTATCTTGTGGACGATGACGTTATGCAGAGGATAAAAGAGGG‑TGTCTTCCGTACTCGCGAAGAGATGGAAGAGTATCGCCATAGTCGCCTTCAAGTGGGCGCTAAGGTATACGCTGAGCAGTTCGGCATCGACCCTGAGGACGTTGATTATCAGCGTGGTTTCAACGGGGACATTACCGAGCGTAACATCTCGCTGTATGGTGCGCATGATAACTTCTTGAGCCAGCAAGCTCAGAAGGGCGCTATCATGAACAGCCGAGTGGAACTCAACGGTGTCCTTCAAGAccc                                           >  1:122873/1‑301 (MQ=255)`  
                                                                                                                                                                                                                                                                   `gTCGTAACGCTGCGTATCTTGTGGACGATGACGTTATGCAGAGGATAAAAGAGGG‑TGTCTTCCGTACTCGCGAAGAGATGGAAGAGTATCGCCATAGTCGCCTTCAAGAGGGCGCTAAGGTATACGCTGAGCAGTTCGGCATCGACCCTGAGGACGTTGATTATCAGCGTGGTTTCAACGGGGACATTACCGAGCGTAACATCTCGCTGTATGGTGCGCATGATAACTTCTTGAGCCAGCAAGCTCAGAAGGGCGCTATCATGAACAGCCGAGTGGAACTCAACGGTGTCCTTCAAGAcct                                           >  1:177274/1‑300 (MQ=255)`  
                                                                                                                                                                                                                                                                   `gTCGTAACGCTGCGTAGCTTGTGGACGATGACGTTATGCAGAAGATAAAAGAGGG‑TGTCTTCCGTACTCGCGAAGAGATGGAAGAGTATCGCCATAGTCGCCTTCAAGAGGGCGCTAAGGTATACGCTGAGAAGTTCGGCATCGACCCTGAGGACGTTGATTATCAGCGTGGTTTCAACGGGGACATTACCGAGCGTAACATCTCGCTGTATGGTGCGCATGATAACTTCTTGAGCCAGCAAGCTCAGAAGGGCGCTATCATGAACAGCCGAGTGGAACTCAACGGTGTCCTTCAAGAccc                                           <  1:63375/301‑1 (MQ=255)`  
                                                                                                                                                                                                                                                                         `gtcgtagatgtgtataagagtcagACGTGATGCAGAAGATAAAAGAGGG‑TGTCTTCCGTACTCGCGAAGAGATGGAAGAGTATCGCCATAATCGCCTTCAAGAGGGCGCTAAGGTATACGCTGAGCAGTTCGGCATCGACCCTGAGGACTTTGATTATCAGCGTGGTTTCAACGGGGACATTACCGGGCGTAACATCTCGCTGTATGGTGCGCATGATAACTTCTTGAGCCAGCAAGCTCAGAAGGGCGCTATCATGAACAGCCGAGTGGAACTCAACGGTGTCCTTCAAGACCCTGatat                                     <  1:241733/278‑1 (MQ=255)`  
                                                                                                                                                                                                                                                                           `gCTTCGTATCTTGTGGACGATGACTTTATGCAGAAGATAAAAGAGGG‑TGTCTGCCGTACTCGCGAAGATATGGAATAGTATCGCCATAGTCGCCTTCAAGAGGGCGCTAAGGTATACGCTGAGCAGTTCGGCATCGACCCTGAGGACGTTGATTATCAGCGTGGTTTCAACGGGGACATTACCGAGCGTAACATCTCGCTGTATGGTGCGCATGATAACTTCTTGAGCCAGCAAGCTCAGAAGGGCGCTATCATGAACAGCCGAGTGGAACTCAACGGTGTCCTTCAAGACCCTGATAtgc                                   <  1:9119/301‑1 (MQ=255)`  
                                                                                                                                                                                                                                                                           `gCTGCGTATCTTGTGGACGATGACGTTATGCAGCAGATAAAAGAGGG‑TGTCTTCCGTACTCGCGAAGAGATGGAAGAGTATCGCCATAGTCGCCTTCAAGAGGGCGCTAAGGTATACGCTGAGCAGTTCGGCATCGACCCTGAGGACGTTGATTATCAGCGTGGTTTCAACGGGGACATTACCGAGCGTAACATCTCGCTGTATGGTGCGCATGATAACTTCTTGAGCCAGCAAGCTCAGAAGGGCGCTATCATGAACAGACGAGTGGAACTCAACGGTGTCCTTCAAGACCCTGATAtgc                                   <  1:67394/301‑1 (MQ=255)`  
                                                                                                                                                                                                                                                                              `gCGTATCTTGTTGACGATGACGTTATGCAGCAGATAAAAGAGGG‑TGTCTTCCGTACTCGCGAAGAGATGGAAGAGTATCGCCATAGTCGCCTTCAAGAGGGCGCTAAGGTATACGCTGAGCAGTTCGGCATCGACCCTGAGGACGTTGATTATCAGCGTGGTTTCAACGGGGACATTACCGAGCGTAACATCTCGCTGTATGGTGCGCATGATAACTTCTTGAGCCAGCAAGCTCAGAAGGGCGCTATCATGAACAGCCGAGTGGAACTCAACGGTGTCCTTCAAGACCCTGATAtgctgg                                >  1:11143/1‑300 (MQ=255)`  
                                                                                                                                                                                                                                                                                `gTATCTTGTGGACGATGACGTTATGCAGAAGATAAAAGAGGG‑TGTCTTCCGTACTCGCGAAGAGATGGAAGAGTAACGCCATAGTCGCCTTCAAGAGGGCGCTAAGGTATACGCTGAGCAGTTCGGCATCGACCCTGAGGACGTTGATTATCAGCGTGGTTTCAACGGGGACATTACCGAGCGTAACATCTCGCTGTATGGTGCGCATGATAACTTCTTGAGCCAGCAAGCTCAGAAGGGCGCTATCATGAACAGCCGAGTGGAACTCAACGGTGTCCTTCAAGACCCTGATATGCTGcgt                              <  1:234790/301‑1 (MQ=255)`  
                                                                                                                                                                                                                                                                                   `tCTTGTGGACGATGACGTTATGCAGCAGATAAAAGAGGG‑TGTCTTCCGTACTCGCGAAGAGATGGAAGAGTATCGCCATAGTCGCCTTCAAGAGGGCGCTAAGGTATACGCTGAGCAGTTCGGCATCGACCCTGAGGACGTTGATTATCAGCGTGGTTTCAACGGGGACATTACCGAGCGTAACATCTCGCTGTATGGTGCGCATGATAACTTCTTGAGCCAGCAAGCTCAGAAGGGCGCTATCATGAACAGCCGAGTGGAACTCAACGGTGTCCtgtctcttctacacatctccgagccc                           >  1:333956/1‑276 (MQ=255)`  
                                                                                                                                                                                                                                                                                    `cTTGTGGACGATGACGTTATGCAGAGGATAAAAGAGGG‑TGTCTTCCGTACTCGCGAAGAGATGGAAGAGTATCGCCATAGTCGCCTTCAAGAGGGCGCTAAGGTATACGCTGAGCAGTTCGGCATCGACCCTGAGGACGTTGATTATCAGCGTGGTTTCAACGGGGACATTACCGAGCGTAACATCTCGCTGTATGGTGCGCTTGATAACTTCTTGAGCCAGCAAGCTCAGAAGGGCGCTATCATGAACAGCCGAGTGGAACTCAACGGTGTCCTTCAAGACCCTGATATGCCGCgtcgtc                          >  1:103775/1‑301 (MQ=255)`  
                                                                                                                                                                                                                                                                                         `ggACGATGACGTTATGCAGAAGATAAAAGAGGG‑TGTCTTCCGTACTCGCAAAGAGATGTAAGAGGATCGCCATAGTCGCCTTAAAGAGGGCGCTAAGGTATACGCTGAGCAGTTCGGCATCGACCCTGAGGACGTTGATTATCAGCATGGTTTCAACGGGGACATTACCGAGCGTAACATCTCGCTGTATGGTGCGCATGATAACTTCTTGAGCCAGCAAGCTCAGAAGGGCGCTATCATGAACAGCCGAGTGGAACTCAACGGTGTCCTTCAAGACCCTGATATGCTGCGTCGTCCAGAc                     <  1:120870/301‑1 (MQ=255)`  
                                                                                                                                                                                                                                                                                           `cagagtaCGTTATGCAGAAGATAATAGAGGG‑TGTTGTCCGGACTCGCGAAGGGATGGAAGAGTATCGCCATAGTCGACTTCAAGAGGGCGCTAAGGTAGACGCTGAGCAGTTCGGCATCGACCCTGAAGACATTGATTATCAGAGTGGTTTCAACGGGTACATTACCGAGCGTAACATCTCGCTTTATGGTGCGCGTGATAACTTCTTGAGCCAGCAAGCTCAGAAGGGCGCTATCATGAAAAGCCGAGTGGAACTCAACGGTGTCCTTAAAGACCCTGATATGCTGCGTCGTCCAGActc                   <  1:315308/295‑1 (MQ=255)`  
                                                                                                                                                                                                                                                                                            `cGATGACGTTATGCAGCAGATAAAAGAGGG‑TGTCTTCCGTACTCGCGAAGAGATGGAAGAGTATCGCCATAGTCGCCTTCAAGAGGGCGCTAAGGTATACGCTGAGCAGTTCGGCATCTACCCTGAGGACGTTGATTATCAGCGTGGTTTCAACGGGGACATTACCGAGCGTAACATCTCGCTGTATGGTGCGCATGATAACTTCTTGAGCCAGAAAGCTCAGAAGGGCGCTATCATGAACAGCCGAGTGGAACTCAACGGTGTCCTTCAAGACCCTGATATGCTGCGTCGTCCAGActct                  >  1:47741/1‑301 (MQ=255)`  
                                                                                                                                                                                                                                                                                              `aTGACGTTATGCAGAGGATAAAAGAGGG‑TGTCTTCCGTACTCGAGAAGAGATGGAAGAGTATCGCCATAGTCGCCTTCAAGAGGGCGCTAAGGTATACGCTGAGCAGTTCGGCATCGACCCTGAGGACGTTGATTATCAGCGTGGTTTCAACGGGGACATTACCGAGCGTAACATCTCGCTGTATGGTGCGCATGATAACTTCTTGAGCCAGCAAGCTCAGAAGGGCTCTATCCTGAACAGCCGAGTGGAACTCAACGGTGTCCTTCAAGACCCTGATATGCTGCGTCGTCCAGACTctgc                >  1:34148/1‑301 (MQ=255)`  
                                                                                                                                                                                                                                                                                                    `ttATGCAGCAGATAAAAGAGGG‑TGTCTTCCGTACTCGCGAAGAGATGGTAGAGTATCGCCATAGTCGCCTTCAAGAGGGCGCTAAGGTATACGCTGAGCAGTTCGGCATCGACCCTGAGGACGTTGATTATCAGCGTGGTTTCAACGGGGACATTACCGAGCGTAACATCTCGCTGTATGGTGCGCATGATAACTTCTTGAGCCAGCAAGCTCAGAAGGGCGCTATCATGAACAGCCGAGTGGAACGCAACGGTGTCCTTCAAGACCCGGATATGCTGCGTCGTCCAGACTCTGCTGActt          >  1:515/1‑301 (MQ=255)`  
                                                                                                                                                                                                                                                                                                        `gcagcaGATAAAAGAGGG‑TGTCTTCCGTACTCGCGAAGAGATGGAAGAGTATCGCCATAGTCGCCTTCACGAGGGCGCTAAGGTATACGCTGAGCAGTTCGGCATCGACCCTGAGGACGTTGATTATCAGCGTGGTTTCAACGGGGACATTACCGAGCGTAACATCTCGCTGTATGGTTCGCATGATAACTTCTTGAGCCAGCAAGCTCAGAAGGGCGCTATCATGAACAGCCGAGTGGAACTCAACGGTTTCCTTCAAGACCCCGCTATGCTGCGTCGTCCAGACTCTGCTGActtctgt      >  1:183650/1‑299 (MQ=255)`  

                                                                                                                                                                                                                                                                                                            `|`                                                                                                                                                                                                                                                                                                               
`GGAGGTATGGGCTATCGTGCAGCAACCACTCAGGCCGAACAGCCAAGGTCAAGCCTATTGGACACCATTGGTCGGTTCGCTAAGGCTGGTGCCGATATGTATACCGCTAAGGAACAACGAGCACGAGACCTAGCTGATGAACGCTCTAACGAGATTATCCGTAAGCTGACCCCTGAGCAACGTCGAGAAGCTCTCAACAACGGGACCCTTCTGTATCAGGATGACCCATACGCTATGGAAGCACTCCGAGTCAAGACTGGTCGTAACGCTGCGTATCTTGTGGACGATGACGTTATGCAGAAGATAAAAGAGGG‑TGTCTTCCGTACTCGCGAAGAGATGGAAGAGTATCGCCATAGTCGCCTTCAAGAGGGCGCTAAGGTATACGCTGAGCAGTTCGGCATCGACCCTGAGGACGTTGATTATCAGCGTGGTTTCAACGGGGACATTACCGAGCGTAACATCTCGCTGTATGGTGCGCATGATAACTTCTTGAGCCAGCAAGCTCAGAAGGGCGCTATCATGAACAGCCGAGTGGAACTCAACGGTGTCCTTCAAGACCCTGATATGCTGCGTCGTCCAGACTCTGCTGACTTCTTTGAGA  >  NC_001604.1/28391‑28991`

  

Alignment Legend

Aligned base mismatch/match (shaded by quality score): `ATCG/ATCG` `< 3 ≤` `ATCG/ATCG` `< 10 ≤` `ATCG/ATCG` `< 22 ≤` `ATCG/ATCG` `< 34 ≤` `ATCG/ATCG` `< 38 ≤` `ATCG/ATCG`

Unaligned base: `atcg`    Masked matching base: `atcg`    Alignment gap: `‑`    Deleted base: `‑`
